# Fragmentation of Care Threatens Patient Safety in Peripheral Vascular Catheter Management in Acute Care– A Qualitative Study

**DOI:** 10.1371/journal.pone.0086167

**Published:** 2014-01-14

**Authors:** Enrique Castro-Sánchez, Esmita Charani, Lydia N. Drumright, Nick Sevdalis, Nisha Shah, Alison H. Holmes

**Affiliations:** 1 Centre for Infection Prevention and Management, Imperial College London, London, United Kingdom; 2 Department of Surgery and Cancer and Imperial Centre for Patient Safety and Service Quality, Imperial College London, London, United Kingdom; University of South Australia, Australia

## Abstract

**Background:**

The use of peripheral vascular catheters (PVCs) is an extremely common and necessary clinical intervention, but inappropriate PVC care poses a major patient safety risk in terms of infection. Quality improvement initiatives have been proposed to reduce the likelihood of adverse events, but a lack of understanding about factors that influence behaviours of healthcare professionals limits the efficacy of such interventions. We undertook qualitative interviews with clinical staff from a large group of hospitals in order to understand influences on PVC care behaviors and subsequent patient safety.

**Methods:**

Ten doctors, ten clinical pharmacists, 18 nurses and one midwife at a National Health Service hospital group in London (United Kingdom) were interviewed between December 2010 and July 2011 using qualitative methods. Responses were analysed using a thematic framework.

**Results:**

Four key themes emerged: 1) Fragmentation of management and care, demonstrated with a lack of general overview and insufficient knowledge about expected standards of care or responsibility of different professionals; 2) feelings of resentment and frustration as a result of tensions in the workplace, due to the ambiguity about professional responsibilities; 3) disregard for existing hospital policy due to perceptions of flaws in the evidence used to support it; and 4) low-risk perception for the impact of PVC use on patient safety.

**Conclusion:**

Fragmentation of practice resulted in ill-defined responsibilities and interdisciplinary resentment, which coupled with a generally low perception of risk of catheter use, appeared to result in lack of maintaining policy PVC standards which could reduced patient safety. Resolution of these issues through clearly defining handover practice, teaching interdisciplinary duties and increasing awareness of PVC risks could result in preventing thousands of BSIs and other PVC-related infections annually.

## Introduction

The use of peripheral vascular catheters (PVC) to administer medications is essential in modern healthcare and benefits millions of patients worldwide. One-third of all individuals admitted to hospitals in the National Health Service (NHS) in the United Kingdom (UK) will receive at least one PVC [1], with comparable annual figures in the USA [2]. However, PVC use is not exempt from adverse events for patients, including phlebitis and infection [3], with the incidence of PVC-associated bloodstream infections (PVC-BSI) estimated at ∼0.5 episodes per 1000 catheter-days, or ∼0.1% of all catheters inserted [4]. To place these figures in context, from July 2011-June 2012 there were 15.1 million admissions in English NHS hospitals alone [5], equating to approximately 5 million patients per year with a PVC, 5000 PVC-related BSI, and countless more minor infections. Thus, the importance of optimal PVC management and care as a global health and patient safety issue cannot be underestimated [6].

Within this clinical context, quality improvement initiatives to date have included the use of performance feedback [7], educational programmes [8]–[10] and multimodal ‘bundles’ (e.g. multidisciplinary involvement of ward teams, real-time feedback, senior staff buy-in, surveillance and education) [11], [12]. However, these approaches have achieved limited success, and it has therefore been suggested that the effective adoption of strategies attempting to change healthcare professionals (HCP) practice would benefit from exploring individual [13], social and professional motivations [14], and structural and cultural influences within local organisational networks [15], [16]. Understanding and applying theoretical frameworks from social and psychological sciences has also been proposed to first understand and then address behavioural determinants on this subject [17]. Here we report the findings of a qualitative study to explore the attitudes, beliefs and perceived barriers and facilitators to compliance with recommended best practice around peripheral vascular catheter management and care amongst different healthcare professionals.

## Methods

### Setting

We conducted our study at an English NHS Trust comprising four hospitals and nine satellite clinics with approximately 1500 beds and 9500 staff in London (UK). All the hospitals within the Trust operate under one organizational structure. The organization had showcased innovative infection prevention and control quality improvement initiatives, including hand hygiene education programmes and the use of smartphone applications to support antimicrobial stewardship [18]. In terms of PVC management and care, all hospitals and clinics in the organization are required to follow the Trust policy (Appendix S1), accessible on the hospital computer intranet. The policy is written by a multidisciplinary group of general and specialist doctors and nurses, infection control practitioners, hospital managers and researchers, considering the best available evidence at the time of writing. The policy includes recommendations from national and international guidelines, and it is updated every two years. The Board of Directors of the Trust ratifies and endorses the policy.

### Participant Selection and Recruitment

In line with standard qualitative practice [19], we considered participants from a wide range of specialties to ensure a richness of opinions and experiences whilst eliciting areas of divergent practice. Hospital pharmacists were included in the sample due to their role as medication advisors in the UK, including indications to switch some medications from intravenous to oral form and thus potentially impacting on PVC use. Doctors (physicians and surgeons), nurses, midwives and clinical pharmacists were randomly selected using databases of the Trust’s Human Resources Department. Profession was used to stratify staff. Inclusion criteria required regular patient contact. Reasons for exclusion included limited access to the clinical setting (e.g. laboratory medicine, occupational health and radiology) or leave due to research or maternity at the time of recruitment. Initial invitation emails sent to potential participants were followed up with a reminder after two weeks. £50 compensation (as cash or a donation to charity) was offered to participants. A total of 86 individuals were approached and 39 (45%) agreed to participate. Recruitment continued until thematic data saturation was achieved (see Analysis section below for definition of this criterion).

### Data Collection

Semi-structured, face to face interviews were conducted between December 2010 and July 2011. The majority of the interviews were carried out by two clinical researchers (EC, hospital pharmacist; RE, clinical nurse). Four additional researchers with experience in infectious diseases, clinical psychology, epidemiology and qualitative research (OB, JD, LD, SF) helped conduct the interviews. An interview guide (Appendix S2) with semi-structured interview questions and prompts was developed based on an extensive systematic review [20]. The interviews aimed to explore the attitudes, beliefs and perceived barriers and facilitators to compliance with recommended best practice in PVC management and care for doctors, nurses and pharmacists. All interviewers were briefed in detail on the final interview topic guide, to ensure reliable administration. Interviews lasted approximately 45 minutes (range 17–105 minutes) and took place in locations of participants’ preference and time convenient to them. Interviews were audio recorded and transcribed verbatim, and responses were anonymised before proceeding to the analysis.

### Analysis

An initial thematic framework from five transcripts was developed by two independent researchers using a deductive-inductive approach [21]. The framework was refined and used to analyse the remaining interviews by three researchers. Transcripts were examined line-by-line and coded using thematic analysis with the aid of software (Dedoose, SCRC, 2011). Key categories and themes were identified and iteratively compared within the framework to elicit further emerging codes. Thematic saturation was considered once the continuous collection and analysis of data offered no new information with a redundancy of emerging thematic categories. These categories were used for elaboration of the study findings. Reliability of the analysis was ensured through weekly researcher meetings and discussion of emerging themes until consensus was reached. All researchers agreed to the final major themes.

### Ethics Statement

The research protocol and instruments were approved by the UK National Research Ethics Service. All participants completed written informed consent prior to interview.

## Results

### Characteristics of Participants

Ten doctors (physicians and surgeons), ten clinical pharmacists, 18 nurses and one midwife from a range of specialities and clinical experience participated in the study. Table S1 presents the characteristics of the sample.

### Themes

Four key themes were identified in the analysis: 1) Fragmentation of management and care, demonstrated with a lack of general overview and insufficient knowledge about expected standards of care or responsibility of different professionals; 2) feelings of resentment and frustration as a result of tensions in the workplace, due to the ambiguity about professional responsibilities; 3) disregard for existing hospital policy due to perceptions of flaws in the evidence used to support it; and 4) low risk perception for the impact of PVC use on patient safety.

#### 1) Fragmentation of management and care

Decisions about management of PVCs were highly fragmented, resulting in a disjointed and inefficient practice for staff involved. In such context, fragmentation could be defined as a loss of overview and ownership coupled with professional practice carried out in isolation. Table S2 summarises the responses about responsibility for each step of the process (decision to use catheter; insertion; care and maintenance; decision to remove), illustrating the notion of fragmentation. One nurse described this ambiguity:

‘*I think the responsibilities are quite cloudy, there’s quite a grey area, the doctor will say it’s the nurse and the nurse will say it’s the doctor*’. Nurse, age 36, education.

In highly specialised areas (intensive care unit (ICU) in particular), doctors and nurses were much more adept at identifying what their contribution was:

‘*So the insertion is entirely done by the doctors. And the care is entirely done by the nurses’.* Senior doctor, age 38, PICU.

*‘I think policy is pretty clear for nurses, for anybody’*. Senior nurse, age 51, ICU.

Overall, physicians described themselves as key decision makers regarding PVC use, but had more equivocal ideas regarding follow-up management and care:

*‘I’m not aware of any particularly fixed protocol of what you should do and when you should do it and when you should be concerned about’.* Senior doctor, age 49, oncology.

Nurses, on the other hand, focused on technical tasks (changing dressings, etc.) but shied away from contributing towards decision making. Finally, clinical pharmacists presented themselves as advisors about the medications to be administered (“*As long as I make sure that the medication is appropriate and it has been done the right way*”. Pharmacist, age 25, general services). Expanding their existing role (by recommending the removal of unnecessary PVCs, for example) did not seem interesting:

‘*I don’t feel it’s a pharmacist’s responsibility to advise that a cannula be taken out.*’ Senior pharmacist, age 28, geriatric medicine.

For doctors, the ambiguity about their own responsibilities was reinforced by unspoken expectations that others would fill any gaps in care. For example, if documentation was left unfinished after inserting a PVC, doctors would expect nurses to complete it. The following comments illustrate those unspoken arrangements:

‘*Maybe twice in my life I have* [dated stickers with the date of catheter insertion*]. If I don’t date them then it probably doesn't get done or the nurses will do it.* […] *I'm sure that the nurses have an idea of how long they* [catheters] *have been in’.* Specialist trainee doctor, age 31, renal/general medicine.

‘Q*uite a lot of doctors who insert them don’t fill in the form. So nursing staff will fill that form in for them’.* Senior nurse, age 33, vascular.

Participants realised how such ‘grey areas’ led to inconsistent experiences for patients requiring PVCs, with organizational factors such as the availability of trained professionals influencing the quality of those experiences:

*‘Every day we have someone from 7 am till 3 pm responsible for doing the cannulas* [sic] *and if he’s not around, then it is the doctors and I’m afraid that’s where things do slip’.* Senior nurse, age 44, vascular surgery.

‘*I can imagine on the ward it would be very easy to cut corners a little bit, because you see, they’re very, very short staffed’.* Midwife, age 25, midwifery.

#### 2) Feelings of resentment and frustration

As coping mechanisms against fragmentation and ambiguity, non-confrontational techniques were used to resolve emerging tensions and preserve working relations. For example, staff would prefer to ‘*stand there with a bottle of gel and dispense it into their hands, to hand the doctors gloves and to put the aprons on them*’ (Clinical nurse specialist, age 41, colorectal), rather than asking clinicians to follow the PVC protocol. If similar behavioural ‘buffers’ were insufficient, direct challenges were more likely to occur between individuals known to each other, as otherwise discussing inappropriate practices was avoided altogether:

*‘I would feel awkward challenging somebody I didn’t know. I wouldn’t hesitate if I know people well enough’.* Senior pharmacist, age 50, HIV/sexual health.

However, even asking known colleagues to comply with policy was met with some fear about being labelled ‘*difficult and a nightmare, […] being much easier just to let it go’.* Clinical nurse specialist, age 42, Outpatient Antibiotic Therapy (OPAT) services. Following the policy stipulations rigidly (‘[…] *If they* [catheters] *have to come out they have to come out’.* Nurse, age 36, renal medicine) also increased workloads for doctors and other nurses, resulting in further resentment and frustration:

‘*If people keep pulling lines out and you have so much to do, it's an area of resentment, between doctors and nurses relations, it impacts on your workload’.* Junior doctor, age 28, A&E/ICU.

‘*The problem for them* [doctors] *is it will just be another job on them for things to do, if you take a line out and then they need IV saline or something’.* Senior doctor, age 42, microbiology.

However, workloads were not the only factor fuelling resentment; interest from some nurses to gain competencies required to insert PVCs was not encouraged by the organization, leaving them feeling underutilised and frustrated. Those feelings were more evident amongst nurses required to undergo local competency assessments despite receiving prior PVC insertion training elsewhere. Ultimately, such lack of institutional support would lead to disinterest about PVC activities:

‘*But they don’t encourage us to cannulate, which I’m really cross, because I do* [know how to] *cannulate but I’ll have to go for the training’.* Nurse, age 36, renal medicine.

‘*Insertion on our unit is the doctors because, you know…We tend not to do it anymore so we’re a little bit deskilled’.* Nurse, age 51, ICU.

Also associated with frustration was documentation. For example, the multiple formats (paper, electronic) and locations (nursing records at bedside, medical notes in documentation trolley, electronic records at computer stations) further fuelled the fragmentation and isolation. Even the standardised PVC record sheet, designed as quality improvement aide, was considered superfluous:

*‘Nobody wants another document that goes in the patient notes, nobody reads them, and it’s an absolute waste of time’.* Clinical nurse specialist, age 42, OPAT services.

Any difficulties accessing the documents promoted deviations from policy, highlighting the inefficient inter professional and organizational communication:

*‘One problem* […] *is that the information of what you’re meant to do and the guidelines is hard to access or hard to easily access’.* Senior doctor, age 49, oncology.

Overall, this ‘bureaucratization’ left some participants feeling ‘*absolutely surrounded by guidelines and protocols*’ (Senior doctor, age 51, renal medicine), and concerned about the poor quality of the information collected *(‘filled in on autopilot and completely meaningless’*, same participant).

#### 3) Disregard for hospital policies

Perceived flaws in the evidence used to support the local hospital policy affected its credibility. For participants, better studies were required before they would agree to adopt some of the policy indications. The idea of ‘evidence’ seemed to refer to the output from randomised controlled trials, minimising the validity of other study designs or sources such as expert consensus:

‘*The concept of a maximum period that a peripheral line should stay in is all very well and good, saying 72 hours, if that's the cut-off. But I think it’s a bit arbitrary, I'm not sure that there's any evidence about that’.* Specialist trainee doctor, age 31, renal/general medicine.

‘*I think they like to be shown evidence, basically, especially doctors. And if you don't show them evidence, they won't necessarily do what you say just because you're saying it’.* Senior doctor, age 42, microbiology.

The professional background of those involved in policy formulation would also impact upon the perceptions of its relevance and robustness:

*‘Are there local standards? Things are being written but from one perspective. So it's nurses writing standards to measure other nurses but it's not accepted by other disciplines as well. They're just going to look at it and go ‘yeah, a nurse wrote that so what's that got to do with me?’.* Senior nurse, age 39, ICU.

Applying generic guidelines to specific patients was difficult for clinicians, who would consider patient or clinical characteristics (such as age or oedema) in order to provide individualised care:

‘*Some people just don't have veins’.* Junior doctor, age 28, A&E/ICU.

*‘Sometimes* [changing the line] *it’s not always possible with patients that have very poor vascular access,* […] *very occasionally the line will stay in longer than recommended’.* Senior nurse, age 51, ICU.

#### 4) Low risk perception for impact of PVC use on patient safety

Whether due to ubiquitousness or lack of quantifying how many BSIs they are responsible for, PVCs were considered of low value and modest importance within the clinical tools available:

‘*I don’t think the doctors and nurses consider IV access as that important at all’.* Clinical nurse specialist, age 42, OPAT services.

‘[Using catheters] *it’s both routine and not desperately interesting, all I’m interested in is getting the drug in to the patient* […] *and I don’t really pay much attention to something pretty minutia like this* [catheters]*’.* Senior doctor, age 49, oncology.

Perhaps resulting from these ideas, the perceived risk associated with PVC use was low and any adverse events deemed infrequent and with minor consequences for patients:

*‘The worst infection I've seen it's a bit of cellulitis up the arm. Take the line out, give antibiotics, and it goes away’.* Junior doctor, age 28, A&E/ICU.

Some pharmacists admitted that their involvement with PVCs related to concerns about the cost of intravenous medications administered rather than an interest in supporting policy compliance:

*‘The first thing I am looking at is that they are getting an IV when we have got an oral available. That’s pretty expensive […]’*. Senior pharmacist, age 50, HIV/sexual health.

## Discussion

This is one of few studies describing contextual reasons for suboptimal PVC management, care and documentation in the UK. In contrast to the linear and coherent process described in the policy, the clinical management and care of PVCs appears fragmented and ambiguous, with presumed rather than explicit responsibilities for each professional group. The policy is only partially successful in promoting consistent and effective behaviours, as the low risk of adverse events attributed to PVC and social and contextual interactions and beliefs act as disincentive to engage in recommended practices.

Fragmentation has received increasing recognition as a threat to patient safety [22], but its impact at the ward level or on team relationships has not been explored. Whilst the effect of interprofessional demarcations on the cohesiveness of care has been considered previously [23]–[26], our study further highlights the consequences of lacking awareness about professional responsibilities (own and others’) on each step of the PVC management process. Incomplete PVC documentation can threaten patient safety. Clinical practices that are not standardised lead to uneven experiences for patients. An antagonistic team climate disrupts communication, increasing healthcare professionals’ workload unreasonably [27]. Essentially, the underlying fragmentation of care is likely to result in PVCs unnecessarily inserted, or staying *in situ* for longer than needed, leading to the adverse events already described.

In this sense, our results differ significantly from Johansson et al [28], where nurses considered PVC care to be an exclusive nursing task and had a clear perception of their responsibilities. Interestingly, they reported that routines failed due to a lack of documentation, whereas our participants also failed in their routines but complained instead about excessive documentation requirements. Their study, however, focused on one professional group whilst we present a multidisciplinary perspective.

To resolve the loss of ownership and overview that characterises fragmentation, organizations may develop explicit process maps detailing when and how each professional group should participate on PVC management and care, and construct their policy accordingly. Maintaining such detailed policy could be challenging, though, in view of the constant evolution of competencies and scope of clinical practice (for example, with nurses and pharmacists assuming skills previously held by others [29]). Such evolution of roles may improve the current arrangement which does not satisfy some doctors or nurses.

The sole introduction of a clearer pathway would not be enough to resolve existing frustrations, as suggested by the negative consequences reported by individuals following the policy stipulations closely. In the same way that clinical practice does not take place in isolation, HCPs decision to avoid complying with policy is not accidental and is shaped by social determinants such as the opinion and practice from senior clinicians [30]. Exploring those social components and understanding how clinicians integrate them together with previous personal and work experience seems essential. Furthermore, as a policy is unlikely to have robust evidence applicable to every potential clinical scenario, it may be useful to highlight areas where evidence is lacking or limited and allow clinicians to exercise their clinical judgement and intuition [31]. In our study, the background of those involved in policy making influenced the perceived reliability of policy recommendations, adding a further dimension to consider and suggesting that multidisciplinary participation in policy making (not focused on infection prevention and control practitioners only) could increase perceptions about policy quality and the compliance with its recommendations.

In our study, participants associated the use of PVCs with a low risk of adverse events. However, as PVCs are by far the most commonly used invasive devices, their absolute iatrogenic effect could be similar to other vascular catheters such as central venous catheters [6], [32]. Highlighting this perspective within clinical teaching and orientations may help to increase interest in PVC management and care.

As with all qualitative studies, ours had some limitations. As we were not able to recruit participants from some clinical areas, transferability of the results to those settings may be low. The results achieved may reflect organizational as well as wider national policy contexts and thus may not be generalisable to all hospitals in the UK or elsewhere. Participants may have offered socially acceptable responses to the researchers, however, given their negative opinions about hospital policy and some activities that they were supposed to do, it is likely that they were being candid. The proposed themes and hypotheses should be corroborated using quantitative evaluation.

## Conclusion

In summary, our multidisciplinary qualitative study suggests that, unless PVC patient safety initiatives promote a coherent process with explicit responsibilities for the professionals involved, they are unlikely to reduce the adverse events associated with the use of peripheral vascular catheters.

## Supporting Information

Table S1 **Demographic characteristics of participants.**
(DOCX)

Table S2 **Responsibility for different steps in peripheral vascular catheter (PVC) management and care, as reported by participants.**
(DOCX)

Appendix S1 **Current (abridged) policy for peripheral vascular catheter (PVC) management and care.**
(DOCX)

Appendix S2 **Interview guide with questions about peripheral vascular catheters (PVC).**
(DOCX)
