# Supplementary material for: Fragmentation of Care Threatens Patient Safety in Peripheral Vascular Catheter Management in Acute Care– A Qualitative Study
Source: PLoS One. 2014 Jan 14;9(1):e86167. doi: 10.1371/journal.pone.0086167 (PMC3891872; doi:10.1371/journal.pone.0086167)
Supplement: Table S1 — Demographic characteristics of participants. (DOCX) [file pone.0086167.s001.docx]

**Table S1- Demographic characteristics of participants**

| **Profession** | **Age**  **median (range)** | **Area of work** | **Years qualified median (range)** | **Years in organization median (range)** |
| --- | --- | --- | --- | --- |
| Pharmacists | 30 (25-60) | Neonates, Haematology/bone marrow transplant, Respiratory, Oncology, Geriatrics, Pain, HIV | 7 (2-40) | 4 (2-35) |
| Doctors, Surgeons | 38 (31-51) | Paediatric ICU, Microbiology, Orthopaedic surgery, Accident & Emergency, adult ICU, Paediatric Ambulatory, Renal, Stroke, Geriatrics, Cancer | 10 (1-32) | 2 (1-10) |
| Nurses, Midwives | 40 (25-61) | Paediatric ICU, Education, adult ICU, Theatres, OPAT, Vascular surgery, Cardiology, Outpatients, Orthopaedics, Care of the Elderly, Anaesthetics, Women & Children, Renal, Cardiac Catheterisation | 15 (2-35) | 8 (1-26) |
