# Supplementary material for: Fragmentation of Care Threatens Patient Safety in Peripheral Vascular Catheter Management in Acute Care– A Qualitative Study
Source: PLoS One. 2014 Jan 14;9(1):e86167. doi: 10.1371/journal.pone.0086167 (PMC3891872; doi:10.1371/journal.pone.0086167)
Supplement: Table S2 — Responsibility for different steps in peripheral vascular catheter (PVC) management and care, as reported by participants. (DOCX) [file pone.0086167.s002.docx]

**Table S2- Responsibility for different steps in peripheral vascular catheter (PVC) management and care, as reported by participants**

| **Profession** | **Responsibility for PVC use decision** | **Responsibility for PVC insertion** | **Responsibility for ongoing PVC care and maintenance** | **Responsibility for PVC removal decision** |
| --- | --- | --- | --- | --- |
| Senior pharmacist. Age 28 years. Geriatric medicine. Qualified 5 years. At organization 4 years. | Doctor | Doctor | Nurse | Doctor |
| Lead Pharmacist. Age 30 years. Intensive Care Unit, Pain and Anaesthetics. Qualified 7 years. At organization 6 years. | Doctor | Doctor | Nurse | ------ |
| Pharmacist. Age 28 years. Several locations. Qualified 5 years. At organization 5 years. | ------ | ------ | Nurse | ------ |
| Senior pharmacist. Age 60 years. Neonates/project management; Qualified 40 years. At organization 35 years. | ------ | Nurse | Nurse | ------ |
| Senior pharmacist. Age 36 years. Haematology/bone marrow transplant. Qualified 7 years. At organization 4 years. | ------ | Does not know | Does not know | Does not know |
| Senior pharmacist. Age 40 years. Respiratory medicine. Qualified 18 years. At organization 5 years. | ------ | Does not know | Does not know | Does not know |
| Pharmacist. Age 25 years. Several locations. Qualified 3 years. At organization 3 years. | “Prescriber” | Doctor, nurse | Does not know | ------ |
| Pharmacist. Age 25 years. Several locations. Qualified 2 years. At organization 2 years. | ------ | Doctor looking after patient | Nurses, doctors | ------ |
| Senior pharmacist; Age 50 years. HIV/sexual health. Qualified 30 years. At organization 21 years. |  | Does not know | Does not know | ------ |
| Senior doctor. Age 49 years. Oncology. Qualified 13 years. At organization 6 years. | Doctor | Doctor, nurse | Does not know | Does not know |
| Specialist trainee doctor. Age 31 years. Renal medicine. Qualified 7 years. At organization 3 years. | Doctor | Doctor | Does not know | Doctor |
| Senior Nurse. Age 39 years. Paediatric Intensive Care Unit. Qualified for 19 years. At organization 9 years. | Does not know | Does not know | Does not know | Does not know |
| Nurse. Age 36 years. Education. Qualified 8 years. At organization 2 years. | Doctor | Doctor or nurse | Doctor or nurse | Does not know |
| Senior nurse. Age 51 years. Adult Intensive Care Unit. Qualified 31 years. At organization 26 years. | ------ | Doctor | Nurse | Does not know |
| Nurse. Theatres. Qualified 22 years. At organization 8 months. | Doctor | Doctor | Nurse and doctor | Doctor |
| Clinical nurse specialist. Age 42 years. Outpatient Antibiotic Therapy services. Qualified 14 years. At organization 13 years. | ------ | Nurse | Nurse | Does not know |
| Senior nurse. Age 33 years. Vascular medicine. Qualified 10 years. At organization 10 years. | ------ | Nurse, doctor | “person inserting them” | Nurse, doctor |
| Midwife. Age 25 years. Qualified 2 years. At organization 2 years. | Midwives | Midwives | Does not know | Does not know |
| Pharmacist. Oncology. Qualified 14 years. At organization 3 years. | ------ | Nurse | Nurse and patients |  |
| Senior doctor. Age 38 years. Paediatric ICU. Qualified 10 years. At organization 2 years. | Doctor | Doctor | Nurse | Nurse |
| Senior doctor. Age 42 years. Microbiology. Qualified 19 years. At organization 6 years. | ------ | Doctor | Nurse | ------ |
| Nurse. Age 61 years. Outpatients. Qualified 34 years. At organization 5 years. | ------ | Doctor | Nurse, doctor | Does not know |
| Nurse. Age 39 years. Theatres. Qualified 12 years. At organization 9 years. | Doctor | Doctor | Nurse | Does not know |
| Nurse. Age 48 years. Orthopaedics. Qualified 12 years. At organization 10 years. | Doctor | Doctor | Nurse | Nurses |
| Nurse. Age 45 years. Geriatrics. Qualified 15 years. At organization 11 years. | ------ | Doctor | Nurse | Does not know |
| Clinical nurse specialist. Age 41 years. Colorectal. Qualified 15 years. At organization 8 years. | Doctors | Doctor, nurse | Nurse | Does not know |
| Nurse. Age 39 years. Adult Intensive Care Unit. Qualified 21 years. At organization 2 years. | Doctor | Doctor | Nurse | ------ |
| Nurse. Age 47 years. Anaesthetics Women and Children. Qualified 20 years. At organization 8 years. | ------ | Doctor, nurse | Nurse | ------ |
| Nurse. Age 26 years. Vascular Surgery. Qualified 5 years. At organization 5 years. | Doctor | Phlebotomist | Nurse | Doctor |
| Nurse. Age 36 years. Renal medicine. Qualified 16 years. At organization 1 year. | Doctor | Doctor | Nurse | Doctor |
| Senior nurse. Age 44 years. Vascular Surgery. Qualified 18 years. At organization 10 years. | ------ | Phlebotomist, doctor, nurse | Nurse | Doctor |
| Nurse. Age 55 years. Cardiac Catheterisation Laboratory. Qualified 35 years. At organization 21 years. | ------ | Nurse | Nurse, infection control team | ------ |
| Nurse. Age 29 years. Orthopaedics. Qualified 7 years. At organization years. | ------ | Nurse | Doctor, nurse | ------ |
| Surgeon. Age 38 years. Orthopaedic surgery. Qualified 2 years. At organization 1 year. | Doctor | Doctor | Nurse | Doctor |
| Junior doctor. Age 28 years. Accident & Emergency/Intensive Care Unit. Qualified 2 years. At organization 1 year. | ------ | Doctor | Nurse | Does not know |
| Doctor. Age 37 years. Paediatric Ambulatory. Qualified 12 years. At organization 1 year. | Doctor | Doctor | Nurse | Doctor |
| Doctor. Emergency Assessment Unit/Accident & Emergency. Qualified 1 year. At organization 1 year. | Doctor | Doctor | Doctor, nurse | Does not know |
| Senior doctor. Age 51 years. Renal Medicine. Qualified 32 years. At organization 10 years. | Doctor | Doctor | Nurse | Doctor |
| Doctor. Age 39 years. Stroke and Geriatrics. Qualified 2 years. At organization 2 years. | Doctor | Doctor | Nurse | Doctor |
