# Supplementary material for: Fragmentation of Care Threatens Patient Safety in Peripheral Vascular Catheter Management in Acute Care– A Qualitative Study
Source: PLoS One. 2014 Jan 14;9(1):e86167. doi: 10.1371/journal.pone.0086167 (PMC3891872; doi:10.1371/journal.pone.0086167)
Supplement: Appendix S1 — Current (abridged) policy for peripheral vascular catheter (PVC) management and care. (DOCX) [file pone.0086167.s003.docx]

**Appendix S1- Current (abridged) policy for peripheral vascular catheter (PVC) management and care**

| **Equipment**  Peripheral insertion packs are available containing all the equipment required.  **Skin preparation**  Use single patient applicator with 2% Chlorhexidine in 70% isopropyl alcohol and allow to dry.  **Personal protective equipment**  Hands are to be cleaned immediately before donning and after removing gloves. Aprons & gloves are single-use items and should be removed and discarded immediately after use.  **Hand hygiene**  Decontaminate hands before and after each patient contact using correct hand hygiene procedure.  **Dressing**  Use a sterile, transparent, semi-permeable dressing to allow daily observation of insertion site.  **Safe disposal of sharps**  Sharps container should be available at point of use and should not be overfilled. Do not pass sharps from hand to hand.  **Documentation**  All care, maintenance and problem solving of PVC should be documented in the patient’s medical and nursing notes. Date of insertion and audit form for data collection should be entered into notes. Peripheral vascular catheter dressing should be dated.  Peripheral vascular catheter must be re-sited every 72 hours or sooner if there is evidence of phlebitis. If the cannula is left in for longer than the reason must be documented in the clinical notes and reviewed daily. |
| --- |
