# Supplementary material for: Fragmentation of Care Threatens Patient Safety in Peripheral Vascular Catheter Management in Acute Care– A Qualitative Study
Source: PLoS One. 2014 Jan 14;9(1):e86167. doi: 10.1371/journal.pone.0086167 (PMC3891872; doi:10.1371/journal.pone.0086167)
Supplement: Appendix S2 — Interview guide with questions about peripheral vascular catheters (PVC). (DOCX) [file pone.0086167.s004.docx]

**Appendix S2- Interview guide with questions about peripheral vascular catheters (PVC)**

| Peripheral vascular catheter (line insertion, monitoring and administration of intravenous medications) | Explore perceived infection control issues relating to line insertion and monitoring, including:   Knowledge and understanding   Perceived responsibility for infection control   Challenges for/barriers to infection control   Potential facilitators to infection control |  What, if any, aspects of peripheral vascular catheter (including insertion, monitoring, and/or administration of intravenous medications) management does your job involve?   In your opinion, what are the main infection risks associated with peripheral vascular catheters?  o Probe: Are there any infection prevention measures to be considered when inserting peripheral vascular catheters? Could you describe these?  Are there any circumstances where you would not follow infection control measures whilst inserting a peripheral vascular catheter? Can you provide any examples of these?  o Probe: Are there any infection prevention measures to be considered when administering intravenous medications? Could you describe these?  o Probe: What barriers do you personally face in adhering to infection prevention measures when administering intravenous medications?   Are you aware of any specific standards associated with peripheral vascular catheter insertion, monitoring of peripheral vascular catheters or intravenous drug administration? Could you describe these?  o Probe: Where/how, if at all, have you learned about these standards?  o Probe: How do you keep up to date with any changes to procedures or practice relating to peripheral vascular catheter insertion or care (including intravenous medication administration)? How would you like to be kept up to date with changes?   Who, in your view, has responsibility for managing the insertion and care of peripheral catheter?  o Probe: How clear do you think it is, within your department, where responsibilities lie?   Is there any follow-on care associated with peripheral catheter following insertion?  o Probe: Are there any challenges to performing these? If so, what are they?  o Probe: Organisational challenges?   What do you think could be done to improve compliance with recommended infection control guidelines for insertion and care of peripheral catheter? |
| --- | --- | --- |
